# Re-Optimization of Expansion Work of a Heated Working Fluid with Generalized Radiative Heat Transfer Law

**DOI:** 10.3390/e22070720

**Published:** 2020-06-29

**Authors:** Lingen Chen, Kang Ma, Yanlin Ge, Huijun Feng

**Affiliations:** 1 Institute of Thermal Science and Power Engineering, Wuhan Institute of Technology, Wuhan 430205, China; geyali9@hotmail.com (Y.G.); huijunfeng@139.com (H.F.); 2 School of Mechanical & Electrical Engineering, Wuhan Institute of Technology, Wuhan 430205, China; 3 Unit 92941 of PLA, Huludao 125001, China; delmarco1981@sina.com

**Keywords:** generalized radiative heat transfer law, optimal motion path, maximum work output, elimination method, finite time thermodynamics

## Abstract

Based on the theoretical model of a heated ideal working fluid in the cylinder, the optimal motion path of the piston in this system, for the maximum work output, is re-studied by establishing the changed Lagrangian function and applying the elimination method when the initial internal energy, initial volume, finial volume and the process time are given and generalized radiative heat transfer law between the working fluid and heat bath is considered. The analytical solutions of the intermediate Euler-Lagrange arc with square, cubic and radiative heat transfer laws are taken as examples and obtained. The optimal motion path of the piston with cubic heat transfer law, which is obtained by applying the elimination method, is compared with that obtained by applying the Taylor formula expansion method through numerical example. The comparing result shows that the accuracy of the result which is obtained by applying the elimination method is not affected by the length of time of the expansion process of the working fluid, so this result is more universal.

## 1. Introduction

Finding the optimal configurations of thermodynamic processes and systems under different given optimal objectives is one of the most active research directions of the finite time thermodynamics (FTT) theory [1,2,3,4,5,6,7,8,9,10]. For the system of a heated ideal working fluid (WF) in the cylinder, Refs. [11,12] studied the optimal motion path (MP) of the piston under the maximum work output. In this system, the WF was assumed to be ideal gas and the heat transfer law (HTL) between the WF and heat bath was Newton’s HTL. Refs. [13,14,15,16] used the optimization results obtained in Refs. [11,12] to study the optimal MPs of the piston under the maximum power output [13] and the maximum work output [14] when the power input was given, as well as the optimal operation processes of internal [15] and external [16] combustion engines. In practical process, HTL is not always Newton’s HTL and also obeys other laws, and HTLs will affect the optimal configurations of thermodynamic processes and systems. Ref. [17] studied the optimal MP of the piston of a heated ideal WF in the cylinder with linear phenomenological HTL and obtained the analytical solution. Refs. [18,19] used the optimization results obtained in Ref. [17] to optimize the operation processes of internal [18] and external [19] combustion engines with linear phenomenological HTL. Refs. [20,21,22] studied the optimal MPs of the piston of a heated ideal WF in the cylinder under generalized radiative [20], Dulong–Petit [21] and convective-radiative [22] HTLs, respectively, and obtained the first-order approximate analytical solutions by using the Taylor formula expansion method. Refs. [20,21,22] applied the Taylor formula expansion method to simplify a complex differential equation to a linear equation, obtained the equation set of the system, and solved the problem that the analytical solution could not be obtained for the too complex differential equation. The results obtained in Refs. [20,21,22] have certain theoretical guiding significance. However, the Taylor formula expansion method has its own limitation, and the approximate analytical solution obtained by using the first order Taylor formula expansion method also has limitation. The Taylor formula expansion method is only suitable for the expansion process in which the total process time is very short (for example, the expansion time in Refs. [20,21,22]). Considering time-dependent heat conductance, Chen et al. [23,24] also studied the optimal MPs of the piston of a heated ideal WF in the cylinder under Newton’s [23] and generalized radiative [24] HTLs, respectively. Chen et al. [25] studied the optimal MPs of the piston of a heated ideal WF in the cylinder under generalized convective HTL.

In this paper, on the basis of Refs. [11,12,17,20,21,22], using the elimination method to eliminate the variable V(t) by applying optimal control theory (OCT), the optimal MP of the piston of a heated ideal WF in the cylinder is studied by using the single variable E(t) when the HTL between the WF and heat bath is generalized radiative HTL. The analytical solutions of intermediate arc, with square, cubic and radiative HTLs, will be taken as examples in this paper. Numerical examples of the optimal MP of the piston for cases of cubic HTL, which is obtained by using the elimination method, will be provided in this paper, and will be compared with those obtained by using the Taylor formula expansion method. The research on the effect of HTL on the optimal MP of a heated ideal WF in the cylinder can enrich FTT.

## 2. Modeling

**Figure 1:**
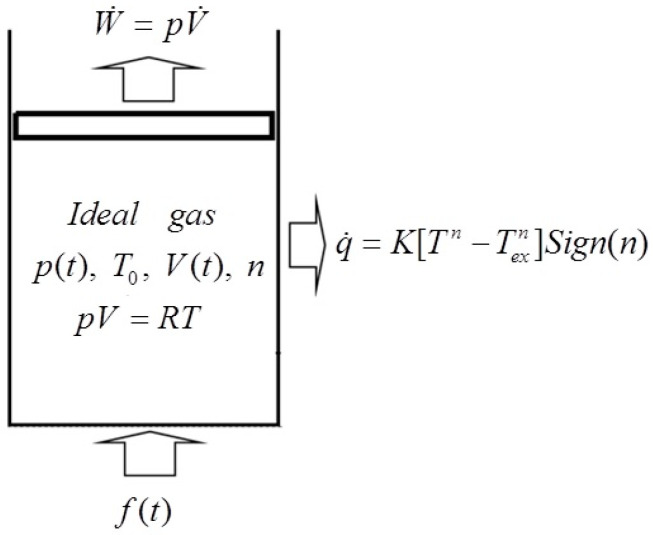
Model diagram of the cylinder with a moveable piston.

Figure 1 shows the model diagram of a cylinder with a moveable piston. In this system, assuming there is 1 mol ideal WF contained in the cylinder, the rate of heat flow f(t) pumped into the cylinder is given, and the HTL between the WF and heat bath is generalized radiative HTL. $q\propto \Delta (T^{n})$ is the heat flow rate through the cylinder wall. K is the heat conductance, $T_{ex}$ and T are the temperatures of the heat bath and WF, respectively, n is the power exponent and Sign(n) is a symbolic function: if n>0, then Sign(n)=1, and if n<0, then Sign(n)=−1. Furthermore, both the inertia impacts of the WF and the piston, and the friction loss of the piston are all ignored.

In this system, the first law of thermodynamics can be written as

(1) $$f\left(t\right)-\dot{E}\left(t\right)-\dot{W}\left(t\right)-Sign\left(n\right)\left[T^{n}\left(t\right)-T_{ex}{}^{n}\right]K=0$$

where W(t) is the work, E is the internal energy, the dot above the variable represents the rate of change of this variable with time.

When the WF in the cylinder is heated, the WF will expand, and the work W produced during this process in the time interval $\left(0,t_{m}\right)$ is

(2) $$W=\int_{0}^{t_{m}}p(t)\dot{V}(t)dt$$

where V and p are the volume and pressure of the WF, respectively. As demonstrated by Ref. [12], the irreversible efficiency η of the process can be written as

(3) $$\eta =W/\left\{RT_{ex}\ln\left[V_{m}/V(0)\right]+E_{p}\right\}$$

where $RT_{ex}\ln[V_{m}/V(0)]$ is the maximum work produced by the WF expanding from V(0) to $V_{m}$ under constant temperature $T_{ex}$, and $E_{p}=\int_{0}^{t_{m}}f(t)dt$ is the total energy added to the WF.

## 3. Optimal Solutions

The general solution is provided first, and three special cases are then provided.

### 3.1. General Solution

As the WF is an ideal gas, the equations $E=C_{V}T$ and pV=RT can also be used, where R is the gas constant, and $C_{V}$ is molar specific heat at constant volume. One can have $p=ER/VC_{V}$ by combining the above two equations. Substituting it into Equation (2) yields

(4) $$W=\int_{0}^{t_{m}}\frac{ER}{C_{V}}\frac{\dot{V}\left(t\right)}{V\left(t\right)}dt$$

Combining Equations (1) and (4) yields

(5) $$W=\int_{0}^{t_{m}}F\left(t\right)dt-\int_{0}^{t_{m}}\left[\dot{E}\left(t\right)+\frac{Sign\left(n\right)K}{C_{V}{}^{n}}E^{n}\left(t\right)\right]dt$$

where $F\left(t\right)=f\left(t\right)+Sign\left(n\right)KT_{ex}{}^{n}$.

As demonstrated by Ref. [20], the optimization problem is

(6) $$\mathrm{maximize}\;W=\int_{0}^{t_{m}}\frac{\dot{V}\left(t\right)E(t)R}{V\left(t\right)C_{V}}dt$$

The constraint condition is Equation (1).

For the above problem, the changed Lagrangian function is established [20]

(7) $$L=\frac{\dot{V}(t)E(t)R}{V(t)C_{V}}+\lambda (t)\left\{\dot{E}(t)-F(t)+\frac{\dot{V}(t)E(t)R}{V(t)C_{V}}+Sign(n){\left[\frac{E(t)}{C_{V}}\right]}^{n}K\right\}$$

The Lagrange multiplier λ(t) in Equation (7) is a function of time.

Solving the Euler-Lagrange (E−L) equation for the problem of Equation (7) gives [20]

(8) $$\dot{E}=\frac{E\dot{F}(t)}{\left(n-1)K{\left(\frac{E}{C_{V}}\right)}^{n}Sign(n)+F(t)(n+1\right)}$$

When n=2, 3 and 4, if the expansion process time is short (for example $t_{m}=0.05$ s), the first-order approximate analytical solution for Equation (8) can be obtained by applying Taylor formula expansion method [20], and the first-order approximate analytical solution is

(9) $$\begin{array}{cc} E(t) & =E^{\prime }(0)+{\dot{E}}^{\prime }(0)t+O(t)\\ & \approx E^{\prime }(0)+\frac{E\dot{F}(t)}{(n+1)F(t)+Sign(n)(n-1){\left(\frac{E}{C_{V}}\right)}^{n}K}t \end{array}$$

In this paper, the elimination method introduced in Appendix B of Ref. [12] is adopted to obtain an analytical solution about the E−L arc. Using the OCT to eliminate the variable V(t), the above optimization problem becomes a one-variable problem, and the optimal MP of the piston can be obtained by the single variable E(t).

Since the MP only depends on the term $\int_{0}^{t_{m}}\left[\dot{E}\left(t\right)+\frac{Sign\left(n\right)K}{C_{V}{}^{n}}E^{n}\left(t\right)\right]dt$ of Equation (5), the optimization problem can be changed to the problem

(10) $$\mathrm{minimize}\;\int_{0}^{t_{m}}\left[E^{}\left(t\right)+\frac{Sign\left(n\right)K}{C_{V}{}^{n}}E^{n}\left(t\right)\right]dt$$

When Equation (1) is divided by E(t), one can have

(11) $$\frac{F\left(t\right)-K{\left[E\left(t\right)/C_{V}\right]}^{n}Sign\left(n\right)-\dot{E}\left(t\right)}{E\left(t\right)}=\frac{\dot{V}\left(t\right)R}{V\left(t\right)C_{V}}$$

Since the values of V(0) and $V_{m}$ are assumed to be given, the constraint of the equivalent optimization problem can be obtained by integrating Equation (11) over time

(12) $$\begin{array}{l} \int_{0}^{t_{m}}\frac{F\left(t\right)-K{\left[E\left(t\right)/C_{V}\right]}^{n}Sign\left(n\right)-\dot{E}\left(t\right)}{E\left(t\right)}dt\\ =(R/C_{V})\ln\left[V_{m}/V\left(0\right)\right]=\mathrm{constant} \end{array}$$

To minimize Equation (10) under the constraint of Equation (12), the modified Lagrangian function is formed as:

(13) $$L=\dot{E}\left(t\right)+\frac{Sign\left(n\right)K}{C_{V}{}^{n}}E^{n}\left(t\right)+\frac{\lambda }{E}\left\{F\left(t\right)-K{\left[E\left(t\right)/C_{V}\right]}^{n}Sign\left(n\right)-\dot{E}\left(t\right)\right\}$$

where λ is the constant Lagrange multiplier. The problem has become the one-variable optimization problem.

The E−L equation for Equation (13) is

(14) $$0=C_{V}^{n}E^{2}(\frac{\partial L}{\partial E}-\frac{d}{dt}\frac{\partial L}{\partial \dot{E}})=nKE^{n+1}-Sign\left(n\right)\left(n-1\right)\lambda KE^{n}-\lambda C_{V}^{n}F$$

Since Lagrange multiplier λ is a constant, it can be obtained by substituting initial values of E(0) and F(0) into Equation (14)

(15) $$\lambda =\frac{nKE^{n+1}\left(0\right)}{Sign\left(n\right)\left(n-1\right)KE^{n}\left(0\right)+C_{V}{}^{n}F\left(0\right)}$$

Substituting λ from Equation (15) into Equation (14) yields

(16) $$\begin{array}{l} E^{n+1}\left(t\right)-E^{n}\left(t\right)\frac{KE^{n+1}\left(0\right)\left(n-1\right)Sign\left(n\right)}{KE^{n}\left(0\right)\left(n-1\right)Sign\left(n\right)+F\left(0\right)C_{V}{}^{n}}\\ -F\left(t\right)\frac{E^{n+1}\left(0\right)C_{V}{}^{n}}{KE^{n}\left(0\right)\left(n-1\right)Sign\left(n\right)+F\left(0\right)C_{V}{}^{n}}=0 \end{array}$$

Solving Equation (16), the analytical solution of E(t) can be obtained. Substituting the analytical solution of E(t) into Equation (1) and integrating, the analytical solution of V(t) can be written as

(17) $$V\left(t\right)={\left[\frac{E}{E\left(0\right)}\right]}^{\frac{C_{v}}{R}}\exp\left[\frac{C_{V}}{R}\int_{0}^{t}\frac{F-Sign\left(n\right)K{\left(\frac{E}{C_{V}}\right)}^{n}}{E}dt\right]V\left(0\right)$$

The optimal process that is determined by Equations (16) and (17) is named E−L arc.

As the same with the results obtained in Ref. [12], one can also conclude that the optimal MP of the piston when the work output is the maximum consists of three segments; this problem is called the linkage problem of OCT. The solution for this problem consists of following three segments: an initial adiabatic process, a middle E−L arc, and a final adiabatic process.

Two items of f(t) and $K[T^{n}\left(t\right)-T_{ex}^{n}]$ are all equal to zero in the adiabatic process; integrating Equation (1), one can obtain

(18) $$E(V)={\left(V/V_{i}\right)}^{-R/C_{V}}E(V_{i})$$

For the initial adiabatic process, assuming the initial values of E(0) and V(0) are given, $E^{\prime }\left(0\right)$ and $V^{\prime }\left(0\right)$ are the final values of internal energy and volume, respectively. The motion equations of the three segments are as follows.

Segment (1) is the adiabatic process of the WF expanding form V(0) to $V^{\prime }\left(0\right)$ at t=0. For this process, one has

(19) $$E^{\prime }(0)=E(0){\left[V(0)/V^{\prime }(0)\right]}^{R/C_{V}}$$

Segment (2) is the E−L arc between t=0 and $t=t_{m}$. In this segment, the WF expands from the initial state [$V^{\prime }\left(0\right)$ and $E^{\prime }\left(0\right)$] at t=0 to $t=t_{m}$. For different HTLs, i.e., n equals to different values, the shapes of E−L arc and the corresponding solution methods are all different. When n=−1, 1, 2 and 3, solving Equation (16), the analytical solutions of E(t) can be obtained, and corresponding E−L arcs can also be obtained. When n equals to other values, the analytical solutions cannot be obtained by Equation (16), and numerical algorithm must be used to obtain the numerical solutions.

Segment (3) is the adiabatic process of WF expanding to final volume $V_{m}$ at $t_{m}$. For this process, one can use

(20) $$E_{m}={\left[V(t_{m})/V_{m}\right]}^{R/C_{V}}E(t_{m})$$

where $E(t_{m})$ and $V(t_{m})$ can be solved by Equations (16) and (17) at time $t_{m}$.

When E(0), V(0) and $V_{m}$ are given, the above linkage problem becomes the one-dimensional optimization problem of expansion work W and $E^{\prime }\left(0\right)$, i.e., solving the optimal final state [$E^{\prime }\left(0\right)$, $V^{\prime }\left(0\right)$] of initial adiabatic expansion to obtain the maximum expansion work W.

Combining Equations (1) and (4), one can obtain

(21) $$W=\int_{0}^{t_{m}}F\left(t\right)dt+E^{\prime }(0)-E_{m}-\frac{Sign\left(n\right)K}{C_{V}{}^{n}}\int_{0}^{t_{m}}E^{n}\left(t\right)dt$$

The maximum expansion work W is a function of the variable $E^{\prime }\left(0\right)$, and solving the equation $dW/dE^{\prime }\left(0\right)=0$, the optimal value of $E^{\prime }\left(0\right)$ can be obtained. Substituting W from Equation (21) into the differential equation $dW/dE^{\prime }\left(0\right)=0$ yields

(22) $$\frac{dE_{m}}{dE^{\prime }(0)}+\frac{d\left[Sign\left(n\right)K\int_{0}^{t_{m}}{\left(E/C_{V}\right)}^{n}dt\right]}{dE^{\prime }(0)}=0$$

The analytical solutions of the E−L arc obtained in this paper for n=1 and n=−1 are the same as those of obtained in Refs. [12,17], and the corresponding numerical examples have been also given in Ref. [20]. Herein, other three cases are provided.

### 3.2. Case of n=2

Substituting n=2 into Equation (16) yields

(23) $$E^{3}\left(t\right)-\frac{{{KE}^{\prime }}^{3}\left(0\right)}{{{KE}^{\prime }}^{2}\left(0\right)+C_{V}{}^{2}F\left(0\right)}E^{2}\left(t\right)-\frac{C_{V}{}^{2}{E^{\prime }}^{3}\left(0\right)}{{{KE}^{\prime }}^{2}\left(0\right)+C_{V}{}^{2}F\left(0\right)}F\left(t\right)=0$$

There are three roots of Equation (23), and the acceptable one is as following

(24) $$E\left(t\right)=\frac{{2A}_{1}K+{2\sqrt[{3}]{2}A_{1}{}^{2}K^{2}/B}_{1}+\sqrt[{3}]{4}B_{1}}{6}$$

where

(25) $$A_{1}=\frac{{E^{\prime }}^{3}\left(0\right)}{{{KE}^{\prime }}^{2}\left(0\right)+C_{V}{}^{2}F\left(0\right)}$$

(26) $$B_{1}={\left[{27A}_{1}C_{V}{}^{2}F\left(t\right)+{2A}_{1}{}^{3}K^{3}+3\sqrt{3}\sqrt{{27A}_{1}{}^{2}C_{V}^{4}F^{2}\left(t\right)+{4A}_{1}{}^{4}C_{V}{}^{2}K^{3}F\left(t\right)}\right]}^{1/3}$$

Substituting Equation (24) into Equation (17) yields

(27) $$V\left(t\right)=V^{\prime }\left(0\right){\left[\frac{E}{E^{\prime }\left(0\right)}\right]}^{-C_{v}/R}\exp\left\{\frac{1}{C_{V}R}\int_{0}^{t}\frac{C_{V}^{2}F-KE^{2}}{E}dt\right\}$$

The E−L arc in stage (2) is determined by Equations (24)–(27).

Substituting n=2 into Equation (5) yields

(28) $$W=\int_{0}^{t_{m}}F(t)dt+E(0)-E_{m}-\frac{K}{C_{V}{}^{2}}\int_{0}^{t_{m}}E^{2}(t)dt$$

Substituting $t=t_{m}$ into Equations (24) and (27) yields

(29) $$E\left(t_{m}\right)=\frac{{2A}_{1}K+2\sqrt[{3}]{2}A_{1}{}^{2}K^{2}/B_{1}^{\prime }+\sqrt[{3}]{4}B_{1}^{\prime }}{6}$$

(30) $$V\left(t_{m}\right)=V^{\prime }\left(0\right){\left[\frac{E\left(t_{m}\right)}{E^{\prime }\left(0\right)}\right]}^{-C_{v}/R}\exp\left\{\frac{1}{C_{V}R}\int_{0}^{t_{m}}\frac{C_{V}^{2}F-KE^{2}}{E}dt\right\}$$

where

(31) $${B^{\prime }}_{1}={\left[{27A}_{1}C_{V}{}^{2}F\left(t_{m}\right)+{2A}_{1}{}^{3}K^{3}+3\sqrt{3}\sqrt{{27A}_{1}{}^{2}C_{V}^{4}F^{2}\left(t_{m}\right)+{4A}_{1}{}^{4}C_{V}{}^{2}K^{3}F\left(t_{m}\right)}\right]}^{1/3}$$

Combining Equations (19), (20), (29) and (30) yields

(32) $$E_{m}=E\left(0\right){\left(\frac{V_{m}}{V\left(0\right)}\right)}^{-R/C_{v}}{\left\{\exp\left[\frac{1}{C_{V}R}\int_{0}^{t_{m}}\frac{C_{V}^{2}F-KE^{2}}{E}dt\right]\right\}}^{R/C_{V}}$$

Taking the derivation of Equation (28) with respect to $E^{\prime }\left(0\right)$ and setting it equal to zero, the optimal value of $E^{\prime }\left(0\right)$ should satisfy the following equation

(33) $$\frac{dE_{m}}{dE^{\prime }\left(0\right)}+\frac{K}{C_{V}{}^{2}}\frac{d\int_{0}^{t_{m}}E^{2}\left(t\right)dt}{dE^{\prime }\left(0\right)}=0$$

### 3.3. Case of n=3

Substituting n=3 into Equation (16) yields

(34) $$E^{4}\left(t\right)-\frac{{{2KE}^{\prime }}^{4}\left(0\right)}{{{2KE}^{\prime }}^{3}\left(0\right)+C_{V}{}^{3}F\left(0\right)}E^{3}\left(t\right)-\frac{C_{V}{}^{3}{E^{\prime }}^{4}\left(0\right)}{{{2KE}^{\prime }}^{3}\left(0\right)+C_{V}{}^{3}F\left(0\right)}F\left(t\right)=0$$

There are four roots of Equation (34), and the acceptable one is as following

(35) $$\begin{array}{l} E\left(t\right)=\frac{1}{2}{\left[A_{{}_{{}^{2}}}^{2}K^{2}-\frac{{2A}_{2}C_{V}^{3}F\left(t\right)}{{\left({3B}_{2}/4\right)}^{1/3}}+{\left(\frac{{2B}_{2}}{9}\right)}^{1/3}\right]}^{1/2}+\frac{A_{2}K}{2}+\frac{1}{2}[{2A}_{2}^{2}K^{2}+\frac{{2A}_{2}C_{V}^{3}F\left(t\right)}{{\left({3B}_{2}/4\right)}^{1/3}}-{\left(\frac{{2B}_{2}}{9}\right)}^{1/3}\\ {+\frac{{2A}_{2}^{3}K^{3}}{{\left[A_{2}^{2}K^{2}-{2A}_{2}C_{V}^{3}F\left(t\right)/{\left({3B}_{2}/4\right)}^{1/3}+{\left({2B}_{2}/9\right)}^{1/3}\right]}^{1/2}}]}^{1/2} \end{array}$$

where

(36) $$A_{2}=\frac{{E^{\prime }}^{4}\left(0\right)}{{{2KE}^{\prime }}^{3}\left(0\right)+C_{V}{}^{3}F\left(0\right)}$$

(37) $$B_{2}=-9A_{2}^{3}C_{V}^{3}K^{2}F\left(t\right)+\sqrt{3}\sqrt{16A_{2}^{3}C_{V}^{9}F^{3}\left(t\right)+27A_{2}^{6}C_{V}^{6}K^{4}F^{2}\left(t\right)}$$

Substituting Equation (35) into Equation (17) yields

(38) $$V\left(t\right)={\left[\frac{E^{\prime }\left(0\right)}{E}\right]}^{C_{V}/R}\exp\left\{\frac{1}{C_{V}^{2}R}\int_{0}^{t}\frac{FC_{V}^{3}-E^{3}K}{E}dt\right\}V^{\prime }\left(0\right)$$

The E−L arc in stage (2) is determined by Equations (35)–(38).

Substituting n=3 into Equation (5) yields

(39) $$W=\int_{0}^{t_{m}}F(t)dt+E(0)-E_{m}-(K/C_{V}{}^{3})\int_{0}^{t_{m}}E^{3}(t)dt$$

Substituting $t=t_{m}$ into Equations (35) and (38) yields

(40) $$\begin{array}{l} E\left(t_{m}\right)=\frac{1}{2}{\left[A_{2}^{2}K^{2}-\frac{2A_{2}C_{V}^{3}F\left(t\right)}{{\left(3B_{{}_{2}}^{\prime }/4\right)}^{1/3}}+{\left(\frac{2B_{{}_{2}}^{\prime }}{9}\right)}^{1/3}\right]}^{1/2}+\frac{A_{2}K}{2}+\\ \frac{1}{2}{\left[\begin{array}{l} 2A_{2}^{2}K^{2}+\frac{2A_{2}C_{V}^{3}F\left(t\right)}{{\left(3B_{{}_{2}}^{\prime }/4\right)}^{1/3}}-{\left(\frac{2B_{{}_{2}}^{\prime }}{9}\right)}^{1/3}+\\ \frac{2A_{2}^{3}K^{3}}{{\left[A_{2}^{2}K^{2}-2A_{2}C_{V}^{3}F\left(t\right)/{\left(3B_{{}_{2}}^{\prime }/4\right)}^{1/3}+{\left(2B_{{}_{2}}^{\prime }/9\right)}^{1/3}\right]}^{1/2}} \end{array}\right]}^{1/2} \end{array}$$

(41) $$V\left(t_{m}\right)=V^{\prime }\left(0\right){\left[\frac{E\left(t_{m}\right)}{E^{\prime }\left(0\right)}\right]}^{\frac{C_{V}}{R}}\exp\left\{\frac{1}{C_{V}^{2}R}\int_{0}^{t_{m}}\frac{C_{V}^{3}F-KE^{3}}{E}dt\right\}$$

where

(42) $$B_{{}_{2}}^{\prime }=-9A_{2}^{3}C_{V}^{3}K^{2}F\left(t_{m}\right)+\sqrt{3}\sqrt{16A_{2}^{3}C_{V}^{9}F^{3}\left(t_{m}\right)+27A_{2}^{6}C_{V}^{6}K^{4}F^{2}\left(t_{m}\right)}$$

Combining Equations (19), (20), (40) and (41) yields

(43) $$E_{m}=E\left(0\right){\left(\frac{V_{m}}{V\left(0\right)}\right)}^{-R/C_{v}}{\left\{\exp\left[\frac{1}{C_{V}^{2}R}\int_{0}^{t_{m}}\frac{C_{V}^{3}F-KE^{3}}{E}dt\right]\right\}}^{R/C_{V}}$$

Taking the derivation of Equation (39) with respect to $E^{\prime }\left(0\right)$, and setting it equal to zero, the optimal value of $E^{\prime }\left(0\right)$, should satisfy the following equation

(44) $$\frac{dE_{m}}{dE^{\prime }(0)}+\frac{K}{C_{V}{}^{3}}\frac{d\int_{0}^{t_{m}}E^{3}(t)dt}{dE^{\prime }(0)}=0$$

### 3.4. Case of n=4

Substituting n=4 into Equation (16) yields

(45) $$E^{5}\left(t\right)-\frac{{{3KE}^{\prime }}^{5}\left(0\right)}{{{3KE}^{\prime }}^{4}\left(0\right)+C_{V}{}^{4}F\left(0\right)}E^{4}\left(t\right)-\frac{C_{V}{}^{4}{E^{\prime }}^{5}\left(0\right)}{{{3KE}^{\prime }}^{4}\left(0\right)+C_{V}{}^{4}F\left(0\right)}F\left(t\right)=0$$

The analytical solution of E(t), with respect to F(t) and $E^{\prime }\left(0\right)$, cannot be obtained because Equation (45) cannot be solved directly. As a result, the method used for cases of n=1, n=−1, n=2 and n=3 cannot be adopted for case of n=4. Such an optimization problem can only be solved numerically.

Substituting n=4 into Equation (5) yields

(46) $$W=\int_{0}^{t_{m}}F(t)dt+E(0)-E_{m}-\frac{K}{C_{V}{}^{4}}\int_{0}^{t_{m}}E^{4}(t)dt$$

## 4. Numerical Example

In this section, only the numerical examples when n=3 are taken as examples and provided.

**Figure 2:**
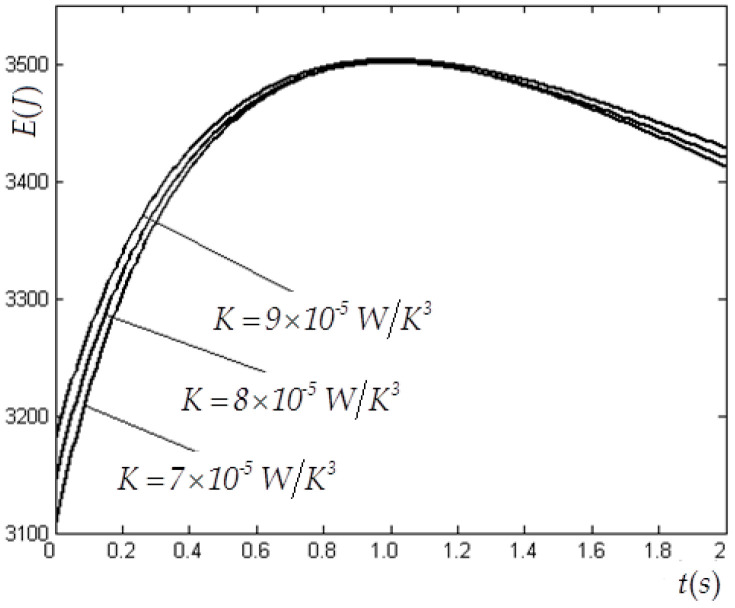
Optimal E versus t obtained by using the elimination method for case of n=3 when $t_{m}=2$ s.

**Figure 3:**
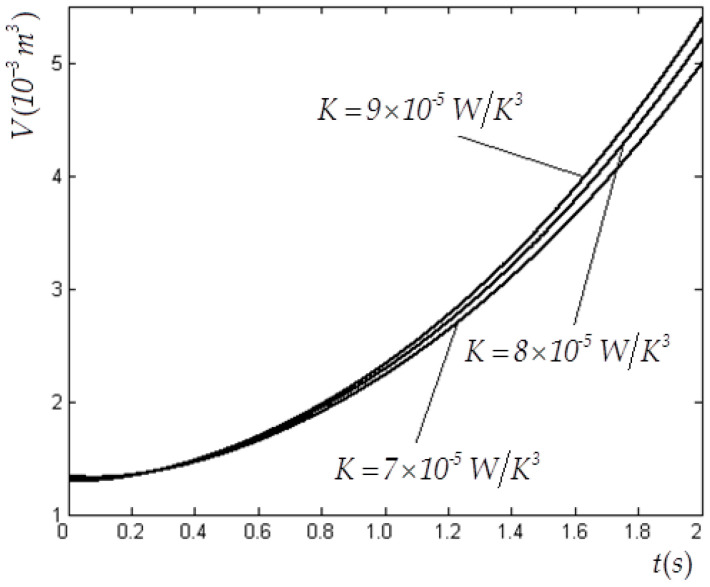
Optimal V versus t obtained by using the elimination method for case of n=3 when $t_{m}=2$ s.

**Figure 4:**
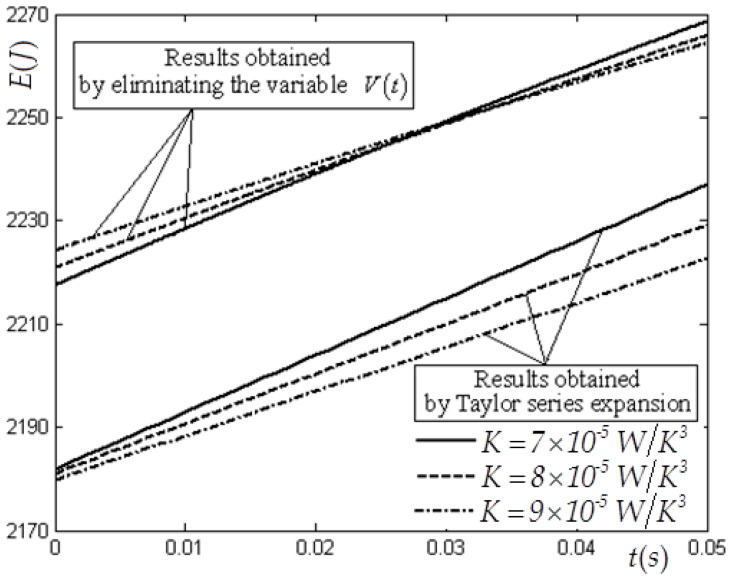
Optimal E versus t obtained by using the elimination and Taylor series expansion methods for case of n=3 when $t_{m}=0.05$ s.

**Figure 5:**
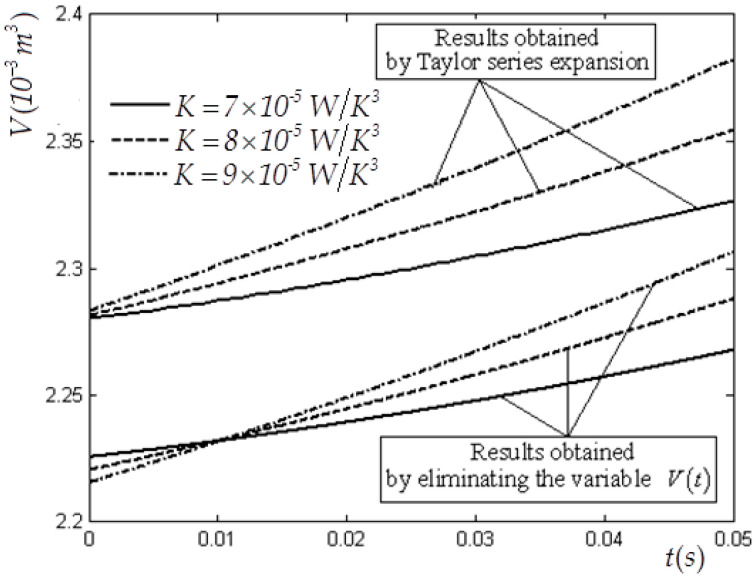
Optimal V versus t obtained by using the elimination and Taylor series expansion methods for case of n=3 when $t_{m}=0.05$.

In this case, $V(0)=1\times 10^{-3}\;$m${}^{3}$, $C_{V}=1.5R$, E(0)=3780 J, $T_{ex}=300\;$K, $V_{m}=8\times 10^{-3}\;$m${}^{3}$ and $f(t)=4200te^{-t}\;$W are selected. Table 1 and Table 2 list the values of the state variables obtained by using the elimination method with variable K for cases of $t_{m}=2$ s and $t_{m}=0.05\;$ s. Table 3 lists the values of the state variables obtained by using the Taylor formula expansion method with variable K for case of $t_{m}=0.05$ s. Figure 2 and Figure 3 show the optimal E and V versus t in the E−L arc obtained by using the elimination method for the case of $t_{m}=2$ s. Figure 4 shows the optimal E versus t in the E−L arc obtained, respectively, by using the elimination and Taylor formula expansion methods for the case of $t_{m}=0.05$ s. Figure 5 shows the optimal V versus t in the E−L arc obtained, respectively, by using the elimination and Taylor formula expansion methods for case of $t_{m}=0.05$ s.

The error percentage of internal energy between results obtained by using the elimination method and those obtained by using the Taylor formula expansion method for case of n=3 is approximately 1.92%, and that of volume is approximately 2.54%.

## 5. Conclusions

Based on the Refs. [11,12,17,20,21,22], using the elimination method to eliminate the variable V(t) by applying OCT, the optimal MP of the piston of a heated ideal WF in the cylinder is studied by the single variable E(t) when the HTL between the WF and heat bath is generalized radiative HTL. The general solution and those for three special cases of n=2, n=3 and n=4 are provided.

Numerical examples obtained by using the elimination method for the optimal MP when n=3 are provided in this paper, and compared with those obtained by using the Taylor formula expansion method. The expansion process time $t_{m}$ has great influences not only on the values of initial $E^{\prime }\left(0\right)$ and $V^{\prime }\left(0\right)$, but also on the optimal MP of the piston. Finally, it can be found that the optimal MPs obtained by using the elimination method are similar to those obtained by using the Taylor formula expansion method when the expansion process time is very short.

The model utilized herein includes only heat transfer loss, without considering friction and the inertia of the piston. Therefore, it is an endoreversible model as those discussed in Refs. [26,27,28,29,30,31,32,33,34,35,36,37]. It can be extended by adding some other dissipations, such as those discussed by Mozurkewich and Berry [38,39] and Hoffmann et al. [40]. Using the elimination method, a more accurate semi-analytical solution is obtained for the optimal MP of the piston in general. The work in this paper can enrich FTT theory.

## Figures and Tables

**Table 1:** Parameters versus K obtained by using the elimination method for case of n=3 when $t_{m}=2$ s.

K (W/K${}^{3}$)	$7\times 10^{-5}$	$8\times 10^{-5}$	$9\times 10^{-5}$
$V^{\prime }(0)\left(10^{-3}m^{3}\;\right)$	1.341	1.316	1.295
$E^{\prime }(0)\left(J\right)$	3108.480	3147.350	3181.910
$V(t_{m})\left(10^{-3}m^{3}\;\right)$	4.9940	5.205	5.388
$E(t_{m})\left(J\right)$	3412.680	3419.810	3428.710
$E_{m}\left(J\right)$	2492.780	2567.670	2634.600
W(J)	4630.820	4661.790	4690.000
η	0.603	0.607	0.611

**Table 2:** Parameters versus K obtained by using the elimination method for case of n=3 when $t_{m}=0.05$ s.

K (W/K${}^{3}$)	$7\times 10^{-5}$	$8\times 10^{-5}$	$9\times 10^{-5}$
$V^{\prime }(0)\left(10^{-3}m^{3}\;\right)$	2.226	2.221	2.216
$E^{\prime }(0)\left(J\right)$	2217.500	2220.850	2224.2000
$V(t_{m})\left(10^{-3}m^{3}\;\right)$	2.2677	2.288	2.306
$E(t_{m})\left(J\right)$	2268.590	2265.820	2264.350
$E_{m}\left(J\right)$	978.929	983.553	988.173
W(J)	2880.230	2886.190	2892.120
η	0.555	0.556	0.557

**Table 3:** Parameters versus K obtained by using the method of Taylor series expansion for case of n=3 when $t_{m}=0.05$ s.

K (W/K${}^{3}$)	$7\times 10^{-5}$	$8\times 10^{-5}$	$9\times 10^{-5}$
$V^{\prime }(0)\left(10^{-3}m^{3}\;\right)$	2.280	2.282	2.284
$E^{\prime }(0)\left(J\right)$	2181.93	2181.100	2179.820
$V(t_{m})\left(10^{-3}m^{3}\;\right)$	2.326	2.355	2.382
$E(t_{m})\left(J\right)$	2237.070	2229.330	2222.680
$E_{m}\left(J\right)$	981.919	986.471	991.195
W(J)	2896.100	2904.8000	2913.4000
η	0.558	0.560	0.561

## Nomenclature

$C_{v}$	Molar heat capacity, J/(mol∙K)
E	Internal energy, J
f	Rate of heated, W
K	Heat conductance, W/K${}^{n}$
L	Modified Lagrangian, W
n	Heat transfer power exponent
p	Pressure, Pa
q	Heat flow rate through the cylinder wall, W
R	Gas constant, J/(mol∙K)
Sign(n)	Sign function
T	Temperature, K
t	Time, s
V	Volume, $m^{3}$
W	Work output, J
Greek symbols	
η	Efficiency
λ	Lagrange multiplier
Subscripts	
ex	External heat bath
m	Final state of expansion process
0	Ambient or reference
